# Rapid auditory and phonemic processing relies on the left planum temporale

**DOI:** 10.21203/rs.3.rs-4189759/v1

**Published:** 2024-04-01

**Authors:** Kelly C. Martin, Andrew T. DeMarco, Sara M. Dyslin, Peter E. Turkeltaub

**Affiliations:** Georgetown University Medical Center; Georgetown University Medical Center, MedStar National Rehabilitation Hospital; Georgetown University Medical Center; Georgetown University Medical Center, MedStar National Rehabilitation Hospital

**Keywords:** lateralization, speech and language, lesion-symptom mapping, stroke and aphasia

## Abstract

After initial bilateral acoustic processing of the speech signal, much of the subsequent language processing is left-lateralized. The reason for this lateralization remains an open question. Prevailing hypotheses describe a left hemisphere (LH) advantage for rapidly unfolding information—such as the segmental (e.g., phonetic and phonemic) components of speech. Here we investigated whether and where damage to the LH predicted impaired performance on judging the directionality of frequency modulated (FM) sweep stimuli that changed within short (25ms) or longer (250ms) temporal windows. Performance was significantly lower for stroke survivors (n = 50; 18 female) than controls (n = 61; 34 female) on FM Sweeps judgments, particularly on the short sweeps. Support vector regression lesion-symptom mapping (SVR-LSM) revealed that part of the left planum temporale (PT) was related to worse performance on judging the short FM sweeps, controlling for performance on the long sweeps. We then investigated whether damage to this particular area related to diminished performance on two levels of linguistic processing that theoretically depend on rapid auditory processing: stop consonant identification and pseudoword repetition. We separated stroke participants into subgroups based on whether their LH lesion included the part of the left PT that related to diminished short sweeps judgments. Participants with PT lesions (PT lesion+, n = 24) performed significantly worse than those without (PT lesion-, n = 26) on stop consonant identification and pseudoword repetition, controlling for lesion size and hearing ability. Interestingly, PT lesions impacted pseudoword repetition more than real word repetition (PT lesion-by-repetition trial type interaction), which is of interest because pseudowords rely solely on sound perception and sequencing, whereas words can also rely on lexical-semantic knowledge. We conclude that the left PT is a critical region for processing auditory information in short temporal windows, and it may also be an essential transfer point in auditory-to-linguistic processing.

## Introduction

Speech is a dynamic auditory signal that encompasses various frequencies and temporal patterns. Embedded within this signal are the basic ‘segmental’ units of language (phonemes) that are strung together to compose more complex elements (e.g., morphemes, words, phrases). While auditory processing is generally performed bilaterally in the brain, including early stages of speech processing, speech and language abilities depend on left hemisphere (LH) perisylvian regions in the majority of adults (Hickok & Poeppel, 2007; Turkeltaub & Coslett, 2010). Thus, there must be a transfer point during speech processing from low-level bilateral auditory processing to left-lateralized linguistic processing.

The stage of processing and the nature of the computations that engender a LH dominance for speech has been a central question in several subfields of neuroscience. Prevailing theories agree that this LH bias is at least partly driven by preferential extraction of rapidly changing auditory information in LH auditory regions (Albouy et al., 2020; Boemio et al., 2005; Flinker et al., 2019; Giroud et al., 2020; Poeppel, 2003; Zatorre et al., 1992; Zatorre, 2022; Zatorre & Belin, 2001). Phonemes are the smallest units perceived in speech, and phonemic variation in the auditory speech signal is carried by changes in the earliest components of the spectrotemporal waveform. Crucially, the spectrotemporal features that differentiate the stop consonants /b/ and /d/ are differentiated by the initial 25ms segment of the second formant. If it sweeps upward, /b/ is perceived; if it sweeps downward, /d/ is perceived.

A stroke to LH perisylvian regions often results in aphasia, a chronic speech and language impairment, which is rarely observed after a right hemisphere (RH) stroke (Dewarrat et al., 2009). In the only study to our knowledge that examined the behavioral interactions between acoustic, phonemic, and phonological processing in adults with LH stroke and aphasia, Kries et al. (2023) found that adults with aphasia exhibited impairments in rise-time discrimination of non-linguistic auditory stimuli (which were all the same duration) as well as shallower categorization slopes in a stop consonant identification task. Additionally, rise-time discrimination predicted outcomes on a behavioral assessment of phonology (Kries et al., 2023).

Further information is needed to determine at which level of speech processing LH regions become critical. Posterior temporal regions have been implicated in auditory-motor integration, and damage to these regions is associated with disrupted speech sound sequencing resulting in phonemic errors (Hickok & Poeppel, 2007). The planum temporale in particular has been characterized as a “computational hub” for the segmenting and template-matching of complex spectrotemporal signals (Griffiths & Warren, 2002). These auditory-motor transformations may include accessing theoretical ‘phonological templates’ (Wise et al., 2001) or ‘sensory representations’ of speech sounds (Hickok & Poeppel, 2007).

In the current study we investigated lesion locations in the LH that cause impairments in processing rapid auditory information, specifically in time windows considered important for speech segmentation. A group of LH stroke (LHS) survivors and healthy controls judged the directionality of short (25ms) and longer (250ms) Frequency Modulated (FM) Sweeps. We used support vector regression lesion-symptom mapping (SVR-LSM) to identify lesion locations that related to diminished task performance, hypothesizing that individuals with a stroke to the left posterior temporal cortex would exhibit worse performance on judging short sweeps in particular. We also examined the behavioral performance of LHS participants with lesions that disrupt their rapid auditory processing on two linguistic assessments of phoneme-level processing: judging the /ba/ versus /da/ identities of stop consonant morphs in a phoneme identification task, and repeating auditorily presented pseudowords (relative to real words). We present key evidence from lesion data about the brain basis of rapid auditory and linguistic processing mechanisms that are thought to engender a LH bias for language abilities.

## Materials and Methods

### Participants

A total of 62 control participants and 59 LHS survivors completed the FM Sweeps, Phoneme Identification, and Pseudoword Repetition tasks. One control participant was excluded because we did not have data on their hearing thresholds. Eight LHS participants were excluded because of neurological factors that impacted the interpretability of their data for our questions (infarcts in both hemispheres (n = 4), LH stroke but right-lateralized language system (n = 1), extensive drug use (n = 1), and significant comprehension deficits that may have impacted the ability to perform the tasks (n = 2). One additional LHS survivor was excluded because we could not be sure that they understood the tasks based on their performance (below-chance mean accuracy on both FM sweep durations, and also on correctly identifying the prototypical stimuli in the phoneme identification task). After these exclusions, 61 control participants and 50 LHS survivors were included in our analyses. See Table 1 for participant characteristics. This study was approved by Georgetown University’s Institutional Review Board, and all participants provided written informed consent.

### Hearing screening

Each participant completed a hearing screening at 500, 1000, 2000, and 4000 Hz (roughly corresponding to the frequency range of the speech signal) in the left and right ears. A standard pure tone audiometry screening was used, involving standard staircasing procedures: the loudness of a tone was stepped up and down until the person could not hear it. A pure tone average (PTA) summarized the threshold (in decibels (dB)) obtained at each of the frequencies tested in each ear. A lower PTA suggests better hearing (the quieter the sound could be while still being heard). PTA values that are 25 dB or lower indicate “normal” hearing in adults at the frequencies tested.

Hearing threshold (i.e., PTA) in each ear were included as a covariate in all analyses. See Table 2 for descriptive statistics on hearing thresholds.

### Experimental Design and Statistical Analysis

The behavioral tasks are available at cognitiverecoverylab.com.

### FM Sweeps Task

#### Stimuli.

The stimuli for the FM Sweeps task consisted of narrow band noise centered at 1000 Hz lasting either 25 or 250ms. This center frequency was linearly modulated with five levels of excursions tested for each of the two stimulus durations. The 250ms sweeps included excursions of 30, 50, 70, 90, and 110 Hz, while the 25ms sweeps included excursions of 150, 200, 250, 300, and 350 Hz. Different excursions were used for the two sweep durations because pilot data revealed that when the excursions were equal, performance on the 250ms sweeps was at ceiling or performance on the 25ms sweeps was at floor. Durations were thus selected for each sweep duration separately based on pilot data to avoid these ceiling and floor effects.

#### Task.

Participants wore over-the-ear Sennheiser HD-579 open-ear headphones and stimuli were presented at a comfortable listening level. Participants were seated at a table in a quiet testing room with a 17” Dell Inspiron touch-screen laptop. The experiment was administered in E-Prime 3.0. Each trial consisted of presentation of one auditory sweep stimulus, followed by a 2AFC button-press of two arrows signifying “up” or “down” on each trial of the task. The task was self-paced and each trial timed out if no response was received after 5 seconds. Each duration×frequency combination was tested using 12 trials, totaling 12×10 = 120 trials. Each trial was randomly presented as a positive frequency excursion or a negative frequency excursion. The task encompassed a total of 2 blocks of 60 trials each. The first block consisted of long sweeps, followed by a block of short sweeps. Each block began with 4 practice trials with feedback on incorrect responses.

#### Statistical analysis.

Mean accuracies for each participant were calculated across all short-sweep and long-sweep trials respectively. We used a two-way repeated-measures ANCOVA to measure the effect of LH Lesion (LHS or Control) and Duration (25ms or 250ms, within-subject variable) on mean accuracy, covarying hearing thresholds in each ear.

### Phoneme Identification Task

#### Stimuli.

We synthesized eight consonant-vowel (CV) syllables by transforming the initial 25ms transition of the second formant from being a fully downward sweep (prototypical /ba/) to being an upward sweep (prototypical /da/) in 10 equal steps. In our final set of eight stimuli, we eliminated the second and penultimate synthesized CV syllables so that the two end-point stimuli were an extra step removed from the 6 middle stimuli, effectively creating unambiguous /ba/ and /da/ end-points.

#### Task.

Participants were seated at a table in a quiet testing room with a touch-screen monitor that prompted the selection of “ba” or “da” after they heard one of the eight CV stimuli, which were presented using the same computer and headphones as the FM Sweeps task. The task included 70 trials with a fixation cross presented for 250ms at the beginning of each trial. Participants completed 4 practice trials with feedback on incorrect responses before beginning the experimental trials. Stimuli 0 and 9, which were the prototypical /ba/ and /da/ CV syllables and were therefore the easiest to correctly judge, were each only presented 5 times, while the other stimuli were each presented 10 times.

#### Categorization Slope.

We calculated the number of times a participant reported a stimulus presentation to be “/ba/” for each stimulus token. The highly replicated pattern of responses for categorical perception is a sigmoid curve that reflects a consistent a affirmative response for half of the stimulus tokens, and then a steep slope that leads to a drop off in that a affirmative response for the other half of the stimuli. This pattern indicates that the participant is perceiving the stimuli as two discrete categories, rather than perceiving the linearly graded change in the acoustic space. The steepness of each participant’s categorization slope represents how abruptly they made the transition in identifying a sound as /ba/ when the stimuli transitioned from being more “/ba/-like” to more “/da/-like.” We fit a sigmoid curve to each participant’s responses averaged on each of the 8 stimulus tokens using a nonlinear least squares approach in the R Statistical Software (v4.1.2; R Core Team 2021; ‘stats’ package). We initialized a, b, and c terms in the model based on where the upper asymptote, the slope at the categorization boundary, and the categorization boundary should fall, respectively, in an idealized version of our sigmoid model (a = 1, b=−0.5, c = 4.5). Since these are negative slopes, a more extreme negative b value indicates a steeper slope, or sharper phoneme category boundary. A b value close to zero indicates a shallow slope, and a positive b value suggests that many responses were incorrectly identified along the category boundary.

#### End-Point Judgments.

We also calculated the difference in each participant’s tendency to identify the first two, most “/ba/-like” stimuli (Stimulus tokens 0 and 2) as /ba/, and the last two, most “/da/-like” stimuli (Stimulus tokens 7 and 9) as /da/. This end-point judgment measure does not rely on curve-fitting procedures that fail to converge for some participants, and provides information about the participant’s certainty about the category membership for the most distinct stimuli, which adds important information beyond the steepness of their categorization slope. For example, a participant could respond /ba/ on only 60% of the most “/ba/-like” stimuli, and continue to respond /ba/ on 40% of the most “/da/-like” stimuli, and still have a steep negative categorization slope if the transition in /ba/ responses happens abruptly for the fifth stimulus. This type of response profile would suggest that a category boundary exists but that the categories are less distinct. Values close to 1 indicate maximum consistency in accurate classification of the end-point stimulus tokens. Values close to 0 indicate equal chance level classification, and negative values indicate a greater number of /ba/ responses to the most “/da/-like” stimuli (i.e., a reversal of categories) and vice versa.

#### Statistical analysis.

We calculated an independent samples t-test with equal variances not assumed to evaluate mean differences between LHS participants and controls on end-point judgments and categorization slope. To evaluate mean differences between LHS participant subgroups, we used one-way ANCOVAs to measure the effect of PT Lesion (+ or −) on the means for end-point judgments and for categorization slope, covarying hearing thresholds and lesion volumes.

### Pseudoword Repetition Task

#### Task.

Participants heard recordings of one-, two-, and three-syllable real words and pronounceable non-words (pseudowords) that matched the real word stimuli on number of syllables and articulatory complexity (see Fama et al., 2019 for more information on stimulus selection). Participants were asked to repeat aloud the word or pseudoword they heard. Each stimulus was followed by a 5-second response period, with additional time provided if necessary. Stimuli were not repeated. Responses were video-recorded for offline scoring.

#### Accuracy.

Responses were scored as correct if the participant produced the target exactly as presented. Any error that was not considered a product of dysarthria or dialectal variation was scored as incorrect.

#### Statistical analysis.

Mean accuracies for each participant were calculated across all syllable lengths for real word and pseudoword trials respectively. To evaluate mean differences between LHS participant subgroups, we used two-way repeated-measures ANCOVAs to measure the effect of LH Lesion (LHS or Control) and Repetition (real words or pseudowords, within-subject variable) on mean repetition accuracy, covarying lesion volume and hearing ability. We measured differences between pseudoword repetition accuracy in controls and LHS participant subgroups using independent samples t-tests. Real word repetition was not examined in controls.

### Behavioral Correlations

We measured whether there was a monotonic relationship between diminished rapid auditory processing abilities and diminished phonological abilities using Spearman’s correlations within each participant group. We examined the relationships between end-point judgments, categorization slope, and pseudoword repetition and the short and long sweeps separately, while controlling for relevant covariates (hearing thresholds in each ear, and lesion volume for stroke participants). All statistical tests were performed using R Statistical Software (v4.1.2; R Core Team 2021).

### Lesion-Symptom Mapping

#### Structural Imaging.

Participants completed a structural scan on a 3T Siemens MAGNETOM Prisma Scanner using a 20-channel head coil at the Georgetown University Center for Functional and Molecular Imaging. High-resolution T1-weighted images were acquired with the following parameters: 176 sagittal slices, voxels = 1 × 1 × 1 mm, matrix = 256 × 256, field of view = 256 mm, GeneRalized Autocalibrating Partially Parallel Acquisition (GRAPPA) = 2, slice thickness = 1 mm, TR = 1900 msec, TE = 2.98 msec. T2-weighted Fluid-Attenuated Inversion Recovery (FLAIR) images were acquired with the following parameters: 192 sagittal slices, voxels = 1 × 1 × 1 mm, matrix = 256 × 256, field of view = 256 mm, slice thickness = 1 mm, TR = 5000 msec, TE = 38.6 msec, TI = 1800 msec, flip angle = 120.

#### Lesion tracing and normalization.

Following the same procedure reported in our other publications (Dickens et al., 2019, 2021; McCall et al., 2022, 2023), lesion masks were manually segmented on each participant’s MPRAGE and FLAIR images using ITK-SNAP software (Yushkevich et al., 2006; http://www.itksnap.org/), and checked by P.E.T., a board-certified neurologist. Native space MPRAGEs and lesion tracings were warped to MNI space using the Clinical Toolbox Older Adult Template as the target template (Rorden et al., 2012) via a custom pipeline. First, brain parenchyma was extracted from each native space image by applying a mask intended to minimize the clipping of gray matter edges. The initial mask was generated by combining the lesion tracing image (binarized) with white and gray matter tissue probability maps generated by the unified segmentation procedure in SPM12 (https://www.fil.ion.ucl.ac.uk/spm/software/spm12/) applied to the original native space image, cost function masked with the lesion tracing. The resulting mask was blurred and inverted to remove nonbrain tissue from the image. The resulting brain extracted image was then normalized using Advanced Normalization Tools software (ANTs; http://stnava.github.io/ANTs/; Avants et al., 2011). Lesion masking was used at each step of the ANTs process. After bias field correction was applied, normalization proceeded using a typical ANTs procedure, including a rigid transform step, an affine transform step, and a nonlinear SyN step. Next, the output of this initial ANTs warp was recursively submitted to three additional applications of the SyN step. Finally, the resulting linear (rigid and affine) and four nonlinear warp fields were concatenated and the original native space MPRAGE and lesion tracings were transformed to the template space using BSpline interpolation. This iterative application of nonlinear warping was intended to improve normalization of expanded ventricles and displaced deep structures in individuals with large lesions. The normalized lesion tracings were finally downsampled to 2.5 mm^3^.

#### Support Vector Regression Lesion Symptom Mapping (SVR-LSM).

We investigated where lesioned voxels predicted lower accuracies on the short FM sweeps trials, controlling for long FM sweeps trial accuracy and lesion volume. These results were visualized on a template MNI brain with MRIcroGL and Mango softwares. SVR-beta-value maps were corrected for multiple comparisons using a continuous family-wise error rate (CFWER) control method. This approach is similar to conventional family-wise error (FWE) multiple comparisons correction but is less conservative (Mirman et al., 2018). Instead of identifying the single top voxel in each random permutation of the data as in conventional FWE methods, the CFWER method takes a parameter, *v*, and identifies the *v*^th^ top voxel in each permutation. This approach is more sensitive for identifying small areas of lesion overlap compared to cluster-based correction methods. This approach is appropriate for our investigations because the hypothesized region of effect (planum temporale) is a small structure. We restricted the analysis to voxels with at least 10% lesion overlap and performed 10,000 permutations with a CFWER threshold of v = 500 cc (i.e., 32 voxels) at a FWER of 0.05. This approach results in a strict voxelwise threshold, for which a lesion symptom map is significant at p < 0.05 if more than 32 voxels survive the threshold. This approach was used to set the voxelwise threshold, and then a standard cluster-level correction was applied at p < 0.05 (based on the same 10,000 permutations) to ensure that surviving voxels were not false positives. Lesion volume was regressed out of both the behavioral measures and the voxelwise lesion data in all analyses.

### PT Lesion Participant Subgroups

We divided the LHS survivors into two subgroups based on whether their lesion territory intersected with the LSM result identified in the FM sweeps analysis. LHS participants were binned into the PT lesion + group if they had any number of voxels shared between their individual lesion mask and a binarized mask of the cluster-corrected LSM result for accuracy on the short sweeps covarying for accuracy on the long sweeps.

## Results

### Rapid auditory processing

#### FM Sweeps Task

##### Behavior.

**Figure 1:**
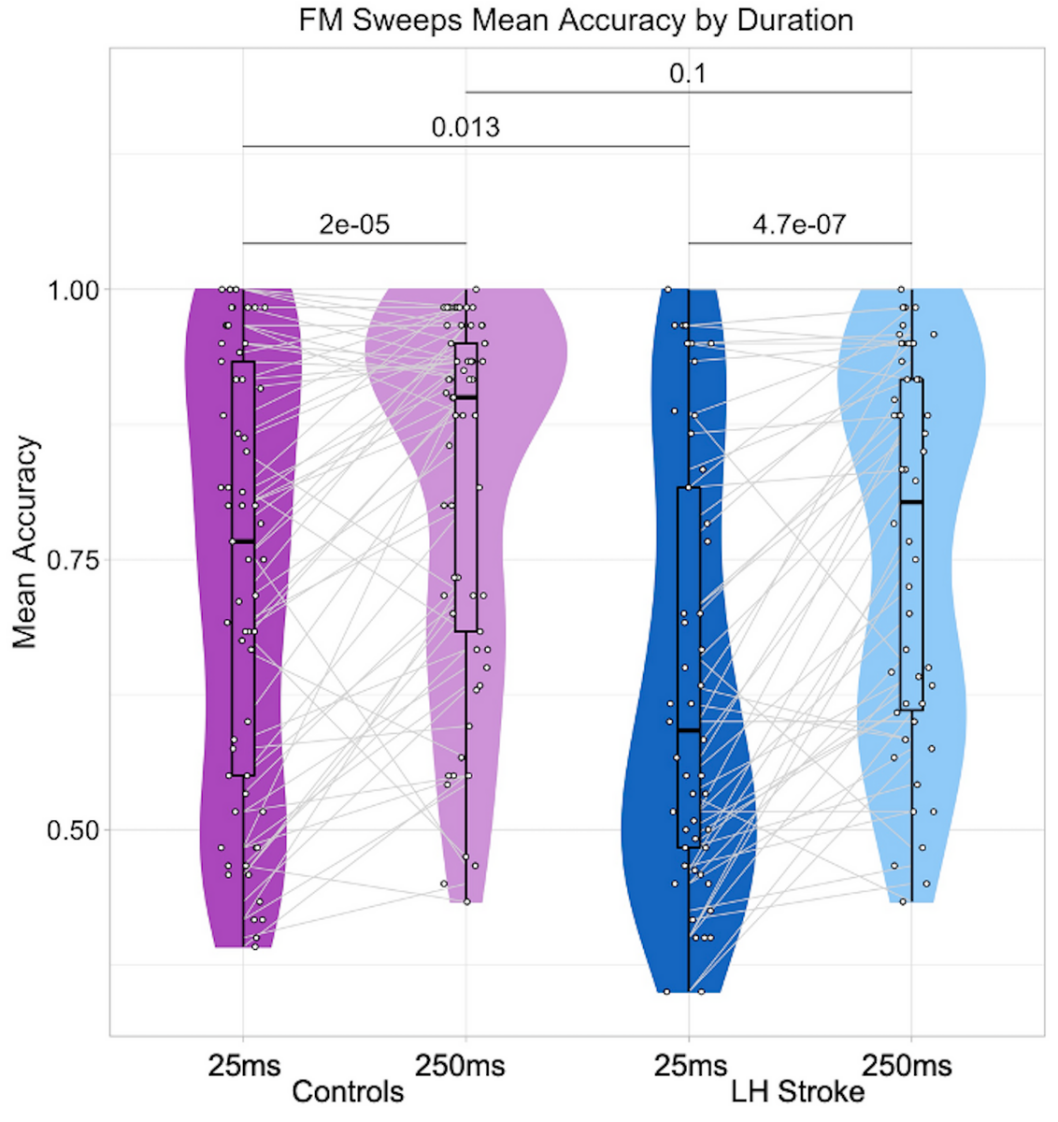
FM sweeps mean accuracy by duration. The average accuracies on responses across trials for the short sweeps (25ms) and long sweeps (250ms) are shown for control participants (dark and light purple, respectively) and LHS participants (dark and light blue, respectively). White dots denote individual participants, and gray lines connect the same individual’s average performance on the short sweeps and the long sweeps. Each embedded boxplot has a bold black line designating the mean accuracy for each duration and participant group. Black lines above the data distributions are labeled with the p-values from two-tailed t-tests (paired t-tests for within-group duration differences, two-sample t-tests for between-group differences for each duration). See Table 2 for descriptive statistics and Table 3 for two-way repeated measures ANCOVA results including hearing as a covariate.

Controls had a higher mean accuracy than LHS participants for both sweep durations (Table 2, Figure 1). Indeed, a two-way repeated measures ANCOVA covarying hearing threshold (Table 3) revealed a significant main effect of LH Lesion on accuracy. There was also a significant main effect of Duration on accuracy, indicating a lower mean accuracy for the shorter sweeps in both groups. We did not expect to measure an interaction effect between LH Lesion and Duration when assessing the full group of LHS survivors, because this result would suggest that strokes to many LH regions, rather than specific locations, produce an impairment on short sweeps judgments. Indeed, we did not measure an interaction effect (Table 3). However, independent samples t-tests revealed that controls performed significantly better than LHS participants on the short sweeps (t(105.2)=2.53, p=0.013), but there were no group differences on the long sweeps (t(103.62)=1.64, p=0.10). This may suggest there is potentially meaningful variability in judging short sweeps among a subset of LHS participants.

##### Lesion-Symptom Mapping.

**Figure 2:**
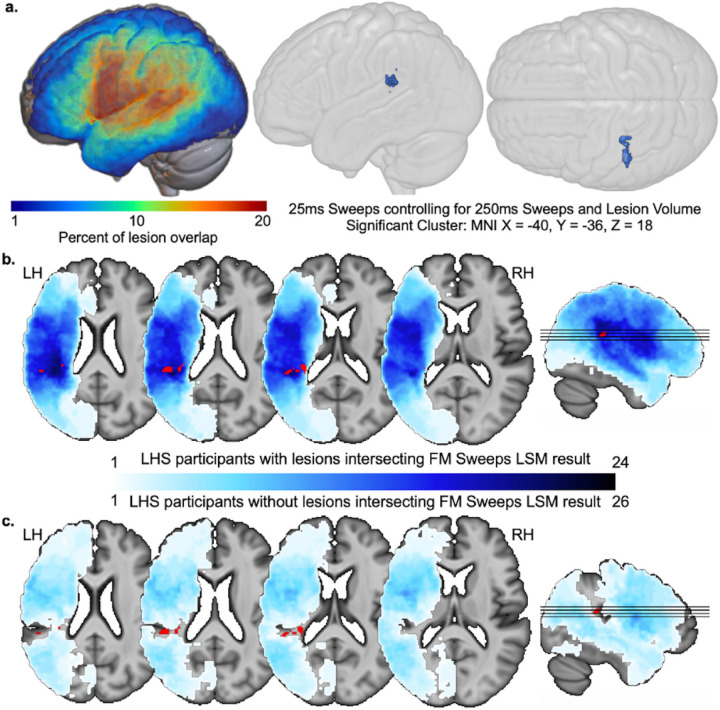
FM sweeps lesion-symptom mapping and creation of PT lesion subgroups. (**a**) Lesion overlap maps are shown on the left for the 50 stroke participants included in the FM sweeps investigation. On the right, 3D-rendered template brains show the lateral and superior views of where lesions related to lower FM sweeps mean accuracy on 25ms trials, covarying for lesion size as well as mean accuracy on the 250ms trials. The center of mass for the cluster surviving CFWER correction is localized to MNI x= −40, y= −36, z= 18 (planum temporale). (**b**) Lesion overlap is shown in blue (greater overlap in darker blue) on select axial and sagittal slices for n=24 LHS participants with lesions that overlap with the FM sweeps LSM result (highlighted in red; same as result in **a**. (**c**) Lesion overlap is shown in the same color scale as **b** for n=26 LHS participants with lesions that do not overlap with the FM sweeps LSM result.

We next performed an SVR-LSM analysis to investigate whether damage to particular parts of the LH predicted a diminished ability to make accurate judgments about the 25ms FM Sweeps stimuli (controlling for performance on 250ms FM sweeps, lesion size, and hearing). A significant cluster was identifed that centered on MNI x= −40, y= −36, z= 18 (within the planum temporale; Figure 2). These results did not change substantially when the same analysis was run without controlling for the 250ms sweep performance. Parallel analyses of the 250ms trials did not reveal any significant results.

### Phonological processing

#### End-Point Judgments.

**Figure 3:**
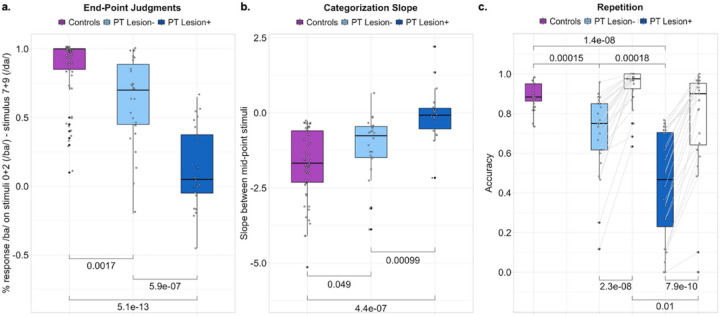
Phonological processing. (a) The average of /ba/ judgments on the end-point stimuli (least ambiguous /ba/ and /da/) are shown for each participant (black circles) in each participant group (left to right: controls in purple, LHS PT lesion− in light blue, and LHS PT lesion+ in dark blue). (**b**) The categorization slope between the mid-point /ba/ and /da/ stimuli was plotted for each participant in each group (same formatting and order as **a**). (**c**) The average repetition accuracy for pseudowords is shown for controls (purple), LHS PT lesion− (light blue), and LHS PT lesion+ (dark blue). Real word repetition accuracies for the LHS subgroups are shown in white. Two-way repeated measures ANCOVAs including LHS participants revealed main effects of PT lesion status and repetition type, and an interaction between these factors, but no effect of lesion volume or hearing ability (see text). For all plots, independent-samples t-tests were used to measure group differences and the brackets are labeled with the associated p-value. Paired samples t-tests were used to compare within-subject pseudoword versus real word repetition accuracies in c. See Table 2 for descriptive statistics.

Phonemes are the most fundamental component of speech, and phoneme segmentation is one of the earliest levels of linguistic analysis performed on the speech signal in the brain. Using a classic phoneme categorization paradigm, we examined whether stop consonant identification was more impaired in stroke participants whose lesions to the left PT were implicated in diminished rapid auditory processing (PT lesion+) as compared to other stroke participants (PT lesion-) and controls. First, we examined how accurately each participant judged the most prototypical /ba/ and /da/ stimuli (the end-points of the stop consonant morph spectrum tested). With judgment abilities scored between −1 (the opposite phoneme identity consistently reported) and 1 (the correct phoneme identity consistently reported), controls scored an average of 0.87 (Table 2), LHS PT lesion− participants scored an average of 0.63, and LHS PT lesion+ participants scored an average of 0.13 (Table 4). Compared to controls, both LHS subgroups performed significantly worse on judging the end-point stimuli (Figure 3a). LHS participants in the PT lesion+ subgroup performed significantly worse than participants in the PT lesion− subgroup (Figure 3a). A one-way ANCOVA measuring the effect of PT Lesion on the mean value of phoneme end-point judgments (Table 5) revealed a significant main effect of PT Lesion but no effect of lesion volume or hearing ability.

#### Categorization slope.

We measured the slope between the two mid-point stop consonant stimuli, which is where the categorization effect is observed (i.e., a change in classifying the stimulus as /ba/ to /da/). Larger negative values reflect more accurate and certain categorization. Statistical comparisons exclude two controls and eight LHS participants (n=2 PT lesion-, n=6 PT lesion+) whose raw responses could not be sigmoid-fit (Supplementary Materials Figure S1). The average slope value was −1.69 for controls (Table 2), −1.15 for LHS PT lesion-, and −0.05 for LHS PT lesion+ (Table 4). Slope values for the LHS PT lesion-subgroup did not statistically differ from controls, but slope values for LHS PT lesion+ subgroup were significantly different from controls and the LHS PT lesion− subgroup (Figure 3b). A one-way ANCOVA measuring the effect of PT lesion on categorization slope (Table 5) revealed a significant main effect of PT lesion and average hearing threshold in the right ear, but no effect of lesion volume or average hearing threshold in the left ear.

#### Pseudoword Repetition.

Repeating pseudowords is the quintessential clinical test of phonological processing in aphasia and is thought to rely on accurate phoneme perception, along with auditory-motor transformations. We next examined whether there was evidence of diminished pseudoword repetition accuracy in PT lesion+ participants relative to PT lesion− participants and controls. Pseudoword repetition accuracy in controls averaged 0.89, which was significantly higher than both LHS subgroups (Figure 3c). In comparing the LHS subgroups, we were interested in determining whether the PT lesion+ subgroup has especially low performance on pseudoword repetition, relative to real word repetition, compared to the PT lesion− subgroup. A two-way repeated measures ANCOVA (Table 5) revealed main effects of PT Lesion and repetition type, and an interaction between PT Lesion and repetition type. Repetition accuracies were lower for pseudowords than real words for both participant subgroups, but the PT lesion+ subgroup performed worse on both pseudoword and real word repetition—and importantly, the PT lesion+ subgroup performed especially poorly on pseudoword repetition relative to real word repetition. There was no effect of lesion volume or hearing abilities, or interaction between either of these covariates and repetition type.

### Behavioral correlations

We examined whether there were any monotonic relationships between the mean accuracies on short and long sweeps judgements and the phonological processing measures in controls and the full group of LHS participants, using partial correlations that accounted for hearing thresholds and lesion volume (LHS only). We did not identify statistically robust relationships in either group (Supplementary Materials, Figure S2 and Table S1).

## Discussion

We found that lesions to the left PT impaired judgments of auditory signals that vary over short time intervals but not longer time intervals. Additionally, we found that stroke participants with lesions to this left PT area were also more impaired on measures of phonological processing relative to stroke participants without such lesions. These subgroup differences were observed while controlling for lesion volume and hearing abilities. We conclude from these findings that the left PT is critically involved in rapid auditory processing in time windows that are relevant for segmenting speech, and damage to this region has a consequential impact on dorsal auditory stream functions that are important for intact speech and language abilities.

Our findings add support for theories on how a LH bias for language processing may arise from a LH bias for rapid temporal processing in non-primary auditory regions (Poeppel, 2003; Zatorre & Belin, 2001). The Asymmetric Sampling in Time (AST) hypothesis (Poeppel, 2001, 2003) in particular predicts a LH bias for temporal integration in 25–50ms windows because formant transitions that differentiate place of articulation unfold in these short windows. Here we tested whether damage to the LH indeed produced a measurable impairment in detecting auditory transitions in short (25ms) but not long (250ms) temporal windows. Stroke survivors as a group performed worse than healthy controls on judging both stimulus durations, which could be attributed to deficits in auditory processing, but also other abilities necessary for making rapid judgments during behavioral tasks. Critically, lesion-symptom mapping revealed that impairments in processing auditory changes in the short but not long temporal windows related to damage to the left PT. In contrast, we did not find any specific lesion locations that resulted in reduced performance on the long sweeps trials. Our findings suggest that there is a LH bias for processing auditory information in short temporal windows, which specifically involves the left planum temporale. When this LH region is damaged, rapid auditory processing is impaired, even when the RH counterpart is intact.

The planum temporale has been implicated in a variety of behaviors related to speech and language processing, as well as spectrotemporal analysis more generally. To unify the various types of processing that the PT has been implicated in, Griffiths and Warren (2002) described the PT as a ‘computational hub’ for spectrotemporal analysis of auditory signals. Within this framework, the PT receives a spectrotemporal sequence that has been somewhat filtered through prior auditory processing stages. Then, through a mechanism that may be similar to independent component analysis (i.e., disentangling components that are mixed together but are presumed independent), it produces separate spectrotemporal components pertaining to the sound object (for further ‘semantic’ analysis in lateral temporal regions) and the sound position (for further spatial analysis in parietal regions; Griffiths & Warren, 2002). In line with this theory, a recent electrocorticography study by Hamilton and colleagues found that the PT and the posteromedial area of Heschl’s gyrus are the first auditory regions to respond (i.e., exhibit high-gamma activity) after the onset of an auditory stimulus, and these regions likely transmit auditory information to other medial and lateral superior temporal regions for subsequent analysis (Hamilton et al., 2021). For some types of highly ecologically relevant input, such as voices, this component separation process may involve template-matching between the incoming sound sequence and previously encountered sequences that may be stored within the PT or in other connected regions. This is in alignment with the description by Wise and colleagues (2001) of phonological template generation in Wernicke’s area to aid in the fast and accurate discrimination of spectrotemporally similar phonemes in speech (Wise et al., 2001).

Here we found that damage to the posterior part of the left PT resulted in impaired judgments of the directionality of frequency-modulated tones, specifically short tones as compared to longer tones, as well as categorizing phonemes that differ based on formant transitions in the same time scale, and also perceiving and repeating an unfamiliar but pronounceable string of phonemes (pseudowords). Under Griffith and Warren’s computational hub framework, damage to the PT should disrupt the level of spectrotemporal processing that would normally produce information about the identity and the spatial localization of a sound. One interpretation of our results, then, is that damage to the PT may disrupt the spectrotemporal decomposition of the acoustic information important for judging the spatial content of the upward- and downward-sweeping FM tones. Under the frameworks described by Griffiths & Warren (2002) and Wise et al. (2001) described above, disrupting this level of analysis may further disrupt the template-matching procedure for phonological discrimination and identity decomposition. This level of phonological disruption may play a role in the pseudoword repetition difficulty we observed in our sample of stroke participants.

However, a disruption in spectrotemporal decomposition after PT damage does not explain why the resulting behavioral impairments may be specific to processing rapid as compared to more slowly-changing auditory information, as we observed. It is unlikely that the PT directly represents the temporal structure of the acoustic stimulus, but rather receives a filtered version of the acoustic information from earlier processing stages (Griffiths & Warren, 2002). Thus, the relationship between damage to the left PT and impaired auditory processing in short temporal windows is not likely explained by damage to neuronal populations within the PT that directly track (e.g., rate filtering) 25ms bins of time. One possibility is that such neuronal populations exist and are damaged in earlier auditory regions, but an impairment in judging FM sweep directionality or phoneme sequencing arises only when the analysis level performed by the PT is damaged. If the PT is intact, perhaps the decomposition process either adjusts or is robust to the loss of information from these earlier neuronal populations that track rapid temporal changes. Indeed, this interpretation is congruent with observations of pure word deafness that arises after bilateral lesions to the posterior superior temporal gyrus: despite intact speech production, reading, and writing, speech sounds are perceived as noise (Poeppel, 2001). Unilateral lesions to the left posterior STG can rarely cause pure word deafness, but a greater number of cases involve bilateral lesions (Griffiths et al., 1999; Poeppel, 2001).

The part of left PT identified in our rapid auditory processing results appears to align with published neuroimaging findings of area Spt (Hickok et al., 2003). According to the dual stream framework proposed by Hickok and colleagues, individuals with damage to area Spt should exhibit impaired production and repetition of low-frequency and novel words relative to common familiar words, and perhaps additional characteristics of conduction aphasia (Buchsbaum et al., 2011). Indeed, we found that stroke participants with lesions to the left PT performed worse on pseudoword repetition than stroke participants without such lesions. Importantly, this PT lesion + subgroup performed even more poorly on pseudoword than real word repetition, which receives additional support from lexical phonological representations and semantic knowledge. Additional research would be needed to determine whether the behavioral effects of lesions to these structures truly dissociate along the functional predictions of the dual and ventral streams.

Our behavioral correlations did not reveal robust relationships between the rapid auditory and phonological processing measures in either group. We found that PT lesions are associated with both rapid acoustic perception and also with the phonological tasks, but in the absence of behavioral correlations we cannot be sure that these abilities (or deficits) are driven by the same cognitive mechanism. A future study should test more partiicpants and use tasks with more trials to confirm whether behavioral relationships exist when the impact of interindividual variability is reduced.

We acknowledge that the size of the PT area used to determine LHS subgroups was very small and that lesion intersection with this small area is based on manually drawn lesion masks which will inherently have a margin of uncertainty. Even though our lesion subgroups are determined by a very small separation in lesion inclusion, the results are robust to lesion volume and hearing abilities, and are present for two different behavioral assessments of phonological processing. We are therefore confident that the results are meaningful, but should be replicated in other samples of LH stroke survivors, and with additional assessments in order to confirm the precise localization of lesion-deficit relationships.

## Conclusions

We investigated whether LH lesions impair processing auditory and linguistic information that unfolds in short temporal windows. We found that stroke survivors with lesions to the left planum temporale were more impaired than stroke survivors without such lesions on tasks that required rapid spectrotemporal analysis. This impairment was measured at multiple levels of early speech analysis, including pure auditory and phonological processing. We conclude that the left PT is a critical region for processing auditory information in short temporal windows, and it may also be an essential transfer point in auditory-to-linguistic processing.

## Figures and Tables

**Table 1:** Participant Characteristics. Demographic information is listed for both groups as well as stroke and aphasia characteristics for left hemisphere stroke (LHS) participants.

	Controls	LHS
**n**	61	50
**Sex**	34 F, 27 M	18 F, 32 M
**Handedness**	58 R, 3 L	45 R, 5 L
**Age**	61.0 (11.7), range: 30.7–83.8	60.0 (11.1), range: 39.4–81.4
**Race**	23 African American, 58 Caucasian	20 African American, 30 Caucasian
**Ethnicity**	59 Non-Hispanic/Latino, 2 unknown	1 Hispanic/Latino, 49 Non-Hispanic/Latino
**Education**	17.0 (2.5), range: 12.0–21.0	16.5 (2.89), range: 9.0–21.0
	**Stroke Type**	41 ischemic, 9 hemorrhagic
	**Stroke Chronicity (years)**	3.9 (4.4), range: 0.12–16.7
	**Western Aphasia Battery Aphasia Quotient**	79.0 (18.7), range: 18.1–98.0

**Table 2.** Descriptive Statistics for Controls and LHS Participants. Performance on the three behavioral tests of interest is listed for each group (LH Lesion 0=Controls, 1=LHS Participants) in terms of number of included data points (n), number of missing data points, mean, standard deviation (Std. Dev.), minimum (Min.) and maximum (Max.). Hearing thresholds in the left (L) and right (R) ear were Pure Tone Average measured in decibels. Frequency Modulated (FM) Sweeps were either 25ms or 250ms duration. Phoneme identification was characterized by end-point judgments or categorization slope. The repetition task included pseudowords (PW) and real words (RW; LHS only). Lesion volume for LHS participants was measured in cubic millimeters (mm^3^).

	FM Sweeps				Phoneme Identification				Repetition				Hearing				Lesion Volume	
	25ms		250ms		End-Points		Slope		PW		RW		L		R			
**LH Lesion**	**0**	**1**	**0**	**1**	**0**	**1**	**0**	**1**	**0**	**1**	**0**	**1**	**0**	**1**	**0**	**1**	**0**	**1**
**n**	61	50	61	50	61	50	59	42	28	50	0	50	61	50	61	50	0	50
**Missing**	0	0	0	0	0	0	2	8	33	0		0	0	0	0	0		0
**Mean**	0.74	0.64	0.82	0.76	0.87	0.39	−1.69	−0.68	0.89	0.57		0.86	25.1	25.5	25.2	23.0		99.57
**Std. Dev.**	0.20	0.20	0.17	0.18	0.22	0.40	1.15	1.15	0.06	0.27		0.22	9.6	10.7	9.9	9.9		85.09
**Min.**	0.39	0.35	0.43	0.43	0.10	−0.45	−5.14	−3.88	0.73	0.00		0.00	7.5	10.0	6.3	6.3		0.97
**Max.**	1.00	1.00	1.00	1.00	1.00	1.00	−0.26	2.21	0.98	0.96		1.00	52.5	61.9	52.5	55.0		349.27

**Table 3.** FM Sweeps Mean Accuracy Group Differences. Results of a two-way repeated measures ANCOVA are shown. We measured the effect of Duration (25ms or 250ms trial type) and LH Lesion (Control or LHS Participant) on sweeps judgment mean accuracy, covarying hearing ability (Pure Tone Average (PTA) measured in decibels). Sum of squares is Type III.

Within Subjects Effects						
	Sum of Squares	df	Mean Square	F	p	p<0.05
Duration	0.102	1	0.102	10.361	0.002	*
Duration * PTA Left Ear	0.005	1	0.005	0.504	0.479	
Duration * PTA Right Ear	0.001	1	0.001	0.145	0.704	
Duration * LH Lesion	0.026	1	0.026	2.672	0.105	
Residuals	1.051	107	0.010			
Between Subjects Effects						
	Sum of Squares	df	Mean Square	F	p	p<0.05
PTA Left Ear	0.009	1	0.009	0.156	0.693	
PTA Right Ear	0.012	1	0.012	0.193	0.661	
LH Lesion	0.318	1	0.318	5.262	0.024	*
Residuals	6.461	107	0.060			

**Table 4.** Descriptive Statistics for LHS Participant Subgroups. Performance on the three behavioral tests of interest is listed for each LHS subgroup (PT lesion 0=lesion does not intersect with short sweeps LSM result, 1=lesion intersects with short sweeps LSM result) in terms of number of included data points (n), number of missing data points, mean, standard deviation (Std. Dev.), minimum (Min.) and maximum (Max.). Hearing thresholds in the left (L) and right (R) ear were Pure Tone Average measured in decibels. Frequency Modulated (FM) Sweeps were either 25ms or 250ms duration. Phoneme identification was characterized by end-point judgments or categorization slope. The repetition task included pseudowords (PW) and real words (RW). Lesion volume was measured in cubic millimeters (mm^3^).

	FM Sweeps				Phoneme Identification				Repetition				Hearing				Lesion Volume	
	25ms		250ms		End-Points		Slope		PW		RW		L		R			
**PT Lesion**	0	1	0	1	0	1	0	1	0	1	0	1	0	1	0	1	0	1
**n**	26	24	26	24	26	24	24	18	26	24	26	24	26	24	26	24	26	24
**Missing**	0	0	0	0	0	0	2	6	0	0	0	0	0	0	0	0	0	0
**Mean**	0.71	0.56	0.81	0.71	0.63	0.13	−1.15	−0.05	0.71	0.43	0.93	0.77	27.1	23.7	24.4	21.5	57.59	145.06
**Std. Dev.**	0.22	0.15	0.18	0.16	0.33	0.29	1.08	0.93	0.20	0.27	0.10	0.27	11.6	9.5	9.1	10.6	50.28	92.33
**Min.**	0.35	0.35	0.47	0.43	−0.20	−0.45	−3.88	−2.17	0.12	0.00	0.63	0.00	10.0	12.5	6.25	10.0	0.97	7.91
**Max.**	1.00	0.95	1.00	0.97	1.00	0.65	0.66	2.21	0.96	0.77	1.00	1.00	61.9	51.3	44.4	55.0	20.06	349.27

**Table 5.** Phonological Processing Group Differences. Three ANCOVA models were run to evaluate mean differences between subgroups of LHS participants covarying hearing ability (Pure Tone Average (PTA) measured in decibels) and lesion volume (cubic mm). The effect of PT Lesion refers to whether a participant’s lesion intersected with the short sweeps LSM result, or did not. Sum of squares is Type III.

*One-way ANCOVA: Effect of PT Lesion on End-Point Judgments*						
	Sum of Squares	df	Mean Square	F	p	p<0.05
PT Lesion	1.783	1	1.783	18.824	< .001	*
PTA Left Ear	0.092	1	0.092	0.967	0.331	
PTA Right Ear	0.245	1	0.245	2.582	0.115	
Lesion Volume	0.053	1	0.053	0.559	0.459	
Residuals	4.262	45	0.095			
*One-way ANCOVA: Effect of PT Lesion on Categorization Slope*						
	Sum of Squares	df	Mean Square	F	p	p<0.05
PT Lesion	10.894	1	10.894	11.664	0.002	*
PTA Left Ear	1.384	1	1.384	1.482	0.231	
PTA Right Ear	4.990	1	4.990	5.343	0.026	*
Lesion Volume	1.134	1	1.134	1.214	0.278	
Residuals	34.559	37	0.934			
*Two-way repeated measures ANCOVA: Effect of PT Lesion and Repetition Type on Repetition Accuracy*						
Within Subjects Effects						
	Sum of Squares	df	Mean Square	F	p	p<0.05
Repetition Type	0.291	1	0.291	23.181	< .001	*
Repetition Type * PTA Left Ear	0.012	1	0.012	0.928	0.340	
Repetition Type * PTA Right Ear	0.005	1	0.005	0.368	0.547	
Repetition Type * Lesion Volume	6.028×10^−4^	1	6.028×10^−4^	0.048	0.828	
Repetition Type * PT Lesion	0.059	1	0.059	4.665	0.036	*
Residuals	0.566	45	0.013			
Between Subjects Effects						
	Sum of Squares	df	Mean Square	F	p	p<0.05
PTA Left Ear	0.006	1	0.006	0.076	0.784	
PTA Right Ear	9.524×10^−4^	1	9.524×10^−4^	0.011	0.916	
Lesion Volume	0.227	1	0.227	2.674	0.109	
PT Lesion	0.440	1	0.440	5.181	0.028	*
Residuals	3.819	45	0.085			
